# Feasibility of Group Problem Management Plus (PM+) to improve mental health and functioning of adults in earthquake-affected communities in Nepal

**DOI:** 10.1017/S2045796020000414

**Published:** 2020-05-26

**Authors:** M. Sangraula, E. L. Turner, N. P. Luitel, E. van ‘t Hof, P. Shrestha, R. Ghimire, R. Bryant, K. Marahatta, M. van Ommeren, B. A. Kohrt, M. J. D. Jordans

**Affiliations:** 1 Transcultural Psychosocial Organization Nepal, Baluwatar, Kathmandu, Nepal; 2 Department of Biostatistics and Bioinformatics and Duke Global Health Institute, Duke University, Durham, USA; 3 Department of Mental Health and Substance Use, World Health Organization, Geneva, Switzerland; 4 University of New South Wales, Sydney, Australia; 5 World Health Organization, Country Office for Nepal, Kathmandu, Nepal; 6 Department of Psychiatry and Behavioral Sciences, George Washington University, Washington, DC, USA; 7 Centre for Global Mental Health, Institute of Psychiatry, Psychology, and Neuroscience, King's College London, London, UK

**Keywords:** Group therapy, mental health, other psychosocial techniques/treatments, randomised controlled trials

## Abstract

**Aims:**

Psychological interventions that are brief, acceptable, effective and can be delivered by non-specialists are especially necessary in low- and middle-income countries, where mental health systems are unable to address the high level of psychosocial needs. Problem Management Plus (PM+) is a five-session intervention designed for those impaired by psychological distress while living in communities affected by adversity. Individual PM+ has demonstrated effectiveness in reducing distress in Kenya and Pakistan, and a group version of PM+ (Group PM+) was effective for conflict-affected women in Pakistan. This paper describes a feasibility and acceptability trial of locally adapted Group PM+ for women and men in an earthquake-affected region of rural Nepal.

**Methods:**

In this feasibility cluster randomised controlled trial, participants in the experimental arm were offered five sessions of Group PM+ and participants in the control arm received enhanced usual care (EUC), which entailed brief psycho-education and providing referral options to primary care services with health workers trained in the mental health Gap Action Programme Intervention Guide (mhGAP-IG). A mixed-methods design was used to assess the feasibility and acceptability of Group PM+. Feasibility was assessed with criteria including fidelity and retention of participants. Acceptability was assessed through in-depth interviews with participants, family members, programme staff and other stakeholders. The primary clinical outcome was depression symptoms assessed using the Patient Health Questionnaire (PHQ-9) administered at baseline and 8–8.5 weeks post-baseline (i.e. after completion of Group PM+ or EUC).

**Results:**

We recruited 121 participants (83% women and 17% men), with equal allocation to the Group PM+ and EUC arms (1:1). Group PM+ was delivered over five 2.5–3 hour sessions by trained and supervised gender-matched local non-specialists, with an average attendance of four out of five sessions. The quantitative and qualitative results demonstrated feasibility and acceptability for non-specialists to deliver Group PM+. Though the study was not powered to assess for effectiveness, for all five key outcome measures, including the primary clinical outcome, the estimated mean improvement was larger in the Group PM+ arm than the EUC arm.

**Conclusion:**

The intervention and trial procedures were acceptable to participants, family members, and programme staff. The communities and participants found the intervention to be beneficial. Because feasibility and acceptability were established in this trial, a fully powered randomised controlled trial will be conducted for larger scale implementation to determine the effectiveness of the intervention in Nepal.

## Introduction

Low- and middle-income countries (LMICs) have fragmented mental health systems which cannot cope with the high level of mental health needs (Jordans and Tol, 2013). LMICs have limited availability to provide adequate mental health treatment (Luitel *et al*., 2015; Thornicroft *et al*., 2017). Innovative psychological treatments that utilise task-sharing are necessary to increase the availability of quality care in LMICs (Patel *et al*., 2018). Problem Management Plus (PM+) is a five-session intervention developed by the World Health Organization (WHO) suitable for low-resource settings for clients impaired by psychological distress (Dawson *et al*., 2015). Randomised controlled trials (RCTs) in Pakistan and Kenya found that PM+ delivered individually is effective for managing practical or psychological problems (Dawson *et al*., 2016; Bryant *et al*., 2017; Khan *et al*., 2019).

A group version of PM+ has been developed with the potential to reach a higher number of people and therefore is more cost-effective for low-resource settings. Group PM+ was shown to be effective in reducing anxiety and depression symptoms in women in a conflict-affected region of Pakistan (Rahman *et al*., 2019). Group PM+ has not yet been evaluated for feasibility and acceptability when delivered in both males and females, nor has it been evaluated following a natural disaster. The aim of this paper is to evaluate the feasibility and acceptability of the Group PM+ intervention in Nepal (Sangraula *et al*., 2018), in order to subsequently conduct a fully powered effectiveness trial of Group PM+.

## Methods

### Setting

Nepal is a low-income country with a history of conflict, political instability and natural disasters. In April 2015, Nepal was hit with two earthquakes resulting in 8000 deaths, 20 000 people injured, damaged homes and livelihood, and substantial internal displacement (Kane *et al*., 2018). Various studies suggest high rates of disabling distress after the earthquakes (Kohrt *et al*., 2012; Luitel *et al*., 2013). An epidemiological study in three districts affected by the earthquake found that one in three adults were experiencing high levels of depression and anxiety symptoms, one in five adults engaged in harmful alcohol use, and one in ten adults had current suicidality (Kane *et al*., 2018).

This Group PM+ feasibility study took place in Sindhuli district, which was impacted by the earthquakes (Sangraula *et al*., 2018). Within Sindhuli district, we selected two Village Development Committees (VDCs) for the intervention and control arms.

### Design

The feasibility study design and *a priori* aims are outlined in a separate pilot and feasibility protocol publication (Sangraula *et al*., 2018), and this study was registered on ClinicalTrials.gov (NCT03359486). The study was designed as a two-arm cluster randomised controlled trial (cRCT), comparing Group PM+ *v*. enhanced usual care (EUC).

### Randomisation

Please see online Supplementary Material for detail.

### Intervention: Group PM+

Participants in the intervention arm received five sessions of Group PM+, with each session lasting 2.5–3 hours. Sessions included: (1) Managing Stress, (2) Behavioural Activation, (3) Managing Problems, (4) Strengthening Social Support and (5) Review of Techniques (Dawson *et al*., 2015). Please see online Supplementary Material for further detail on techniques used in Group PM+.

There were ten groups in the Group PM+ arm. Participants were allocated to groups based on their location of residence. The group consisted of six to eight people separated by gender and with gender-matched facilitators. Volunteer local helpers supported facilitators by organising logistics and reminding participants about the sessions. Community-based psychosocial workers (CPSW) were the service providers for the groups and are a cadre of psychosocial workers in Nepal that are trained through and work for NGOs, such as Transcultural Psychosocial Organization (TPO) Nepal.

### Control: EUC

CPSWs delivered family meetings to participants in both arms. This consisted of: (a) consent to take part in the study and follow-up assessments, (b) psychoeducation on adversity, (c) benefits from support, (d) information on the availability of mental health services by a mental health Gap Action Programme Intervention Guide (mhGAP)-trained health worker in the nearby clinic. After the 2015 earthquakes, the mhGAP Humanitarian Intervention Guide was adapted and contextualised for Nepal (mhGAP HIG). Nepali psychiatrists were taught to train primary care workers using mhGAP (Jordans *et al*., 2016). One health worker from each study VDC received a 10-day mhGAP training to identify, assess and treat common mental disorders (CMDs).

### Main outcomes

The main objective was to determine the acceptability and feasibility of Group PM+ in Nepal, using quantitative and qualitative data. These results will inform changes to the methodology for the fully-powered RCT. The quantitative indicators in Table 1 determined progression to the main trial.

**Table 1.** Feasibility and acceptability criteria and outcomes

Feasibility and acceptability criteria	Definition and measures	Outcomes
Fidelity to Group PM+ elements at the level of 75% or greater	This was operationalised as the mean fidelity checklist for Group PM+ elements across all sessions. A combined competency and fidelity checklist was created based on both Group PM+ elements and common factors in psychological treatments, with the latter items drawn from the ENhancing Assessment of Common Therapeutic factors (ENACT) tool (Kohrt *et al*., 2015*a*, 2015*b*). The tool was used to measure whether or not key activities were implemented and the competency with which they were completed in each session. Clinical supervisors attended at least two of the five sessions per PM+ group and used the fidelity checklist as a tool to rate the skills of the four facilitators. Each session had 9–10 items and rated the facilitator's level of competency to the intervention manual on a scale of 1–3.	All Group PM+ facilitators (*n* = 4) scored ⩾75% in all 5 sessions
Lack of significant socio-demographic group differences	Tabulation of descriptive summaries for baseline characteristics comparing Group PM+ participants and EUC participants without significant group differences in education, economic status, age, gender and medical comorbidities	Participants in both the arms were similar in – age categories (with the mean age around 45–46 years old), gender (16–17% male), occupation (half the participants worked as housewives followed by farming), marital status (around 80% were married followed by 11–15% were widowed) and religion (87–90% practised Hinduism). Participants in the arms differed slightly by their caste group; the intervention arm had a high percentage of Danuwar caste and control arm had a high percentage of Brahman/Chhetri caste. There were also differences in a few other descriptors including most-used language, self-perceived socioeconomic status (SES) and education status.
Retention of at least 67% of participants	Through completion of 5 Group PM+ sessions; 100% retention is defined as attending all five sessions	Of the total participants (*n* = 61), 32 (52.5%) attended all 5 sessions, 14 (23%) attended 4 sessions, 10 (16%) completed 2–3 sessions, 3 (5%) completed 1 session and 2 (3%) did not attend any sessions. 46 (75%) completed 4–5 sessions.
Fewer than 15% missing items	Operationalised as 15% of missing individual items across five key outcome measures (PHQ-9, WHODAS, GHQ, PCL-5 and RTC)	There were no missing outcomes across the five key measurements
Presence of adverse events among fewer than 10% of participants and any serious adverse events	Adverse events included marked increase in suicidal thoughts of trial participants, increased emotional distress and increased family conflict from the start of the trial. Serious adverse events include death of trial participants, suicide attempt, serious violence. This was operationalised as fewer than 10% of participants experiencing any serious adverse events.	A total of seven adverse events (5%) were reported amongst the 121 participants. The majority of these adverse events followed-up by a counsellor included suicidality and included one death due to a health problem unrelated to the study.

### Community detection and case identification

The research assistants (RAs) were briefed on the Community Informant Detection Tool (CIDT), a tool that incorporates vignettes, illustrations and local idioms of distress for lay workers to identify those with CMDs (PPV = 0.68 and NPV = 0.91 for adults) (Jordans *et al*., 2015; Subba *et al*., 2017). While a general distress version was designed to recruit participants for the study, the RAs were trained on the psychosis CIDT so they could identify those that would not qualify for the study. Programme staff used the CIDT to train local leaders, such as female community health volunteers, to identify participants for screening. RAs were informed of potential participants, who were subsequently screened. Please see online Supplementary Material for further information on recruitment and training of non-specialists and RAs.

### Blinding

Please see online Supplementary Material for detail.

### Screening

Please see online Supplementary Material for detail.

### Quantitative assessments

The primary clinical outcome measure was the Patient Health Questionnaire (PHQ-9), which measures symptoms of depression. It has been clinically validated in Nepal with a cut-off score of ⩾10 (sensitivity = 0.94, specificity = 0.80, PPV = 0.42 and NPV = 0.99) (Kohrt *et al*., 2016).

The WHODAS (>16) and the GHQ-12 (>2) were included in the screening as part of the inclusion criteria and as secondary outcome measures (Minhas and Mubbashar, 1996; Tol *et al*., 2009; Tol *et al*., 2010; Thapa and Hauff, 2012). The heart–mind screener, a locally developed tool, was used to determine if participants identified with a local idiom of distress and if they experienced impairment due to these problems (sensitivity = 0.94, specificity = 0.27, PPV = 0.17, NPV = 0.97) (Kohrt *et al*., 2016).

There were two other secondary clinical outcomes that included Post-traumatic Stress Disorder Checklist DSM-5 (PCL-5) and the Psychosocial Mental Health Problems (PMHP). The PCL-5, an eight-item scale, was shown to have comparable diagnostic utility to the 20-item PCL-5 in a recent study (Price *et al*., 2016), and was used to reduce the burden on participants from using the full Nepali version of the PCL (Kohrt *et al*., 2012; Luitel *et al*., 2013). The PMHP scale is a locally developed five-item assessment of common psychosocial problems in Nepal (Luitel *et al*., 2013).

Additionally, The Multidimensional Scale of Perceived Social Support (MSPSS) self-assesses participants' connectedness with family and friends (Zimet *et al*., 1990) and has been locally adapted (Hendrickson *et al*., 2018) and validated to use with Nepali populations (Tonsing *et al*., 2012). The three subscales within the MSPSS were found to be significantly correlated (Family with Friends, *r* = 0.530, *p* < 0.01; Family with Significant Others, *r* = 0.540, *p* < 0.01; and Significant Others with Friends, *r* = 0.575, *p* < 0.01) (Tonsing *et al*., 2012).

The Reducing Tension Checklist (RTC) was developed for this study to evaluate the use of coping strategies of Group PM+ and was developed based on a coping checklist (Neacsiu *et al*., 2010). The items are worded such that participants in the control arm could also endorse these strategies (e.g. questions on helping others, practising slow breathing and tackling everyday problems).

Demographic characteristics were assessed at baseline. Traumatic events were assessed using the Traumatic Events Inventory (TEI) (Schwartz *et al*., 2005), which has been previously used in Nepal (Kohrt *et al*., 2015*c*). An earthquake questionnaire was also developed for this trial to determine the severity in which participants were affected by the earthquake. The Psychological Outcomes Profiles (PSYCHLOPS) (Ashworth *et al*., 2004) was administered pre- and post-intervention and from sessions two to five for the intervention arm to assess the main problems that participants faced. Though the PSYCHLOPS was intended for analysis as a secondary outcome, it was used in the study as a clinical tool for facilitators and clinical supervisors to track the weekly progress of participants. Please see online Supplementary Material for further detail on the timeline of quantitative outcome measures.

### Qualitative evaluation

Qualitative interviews followed a semi-structured interview guide. Key informants included Group PM+ participants (*n* = 7), family members of participants (*n* = 8), Group PM+ facilitators (*n* = 4), CPSWs in the control arm (*n* = 4), control arm participants (*n* = 5) and mhGAP trained health workers (*n* = 2). Both males and females with different rates of retention in the PM+ sessions were interviewed. Focus group discussions were conducted with PM+ participants and programme staff at different time points within the trial. The qualitative interviews explored questions on the acceptability, utility of the intervention, challenges faced and suggestions for trial procedures.

### Data analyses

Quantitative analyses were predominantly descriptive. The main outcomes of interest for this pilot trial were generated using data collected on fidelity, outcome data availability and drop-out. Baseline participant characteristics were summarised by arm. Likewise, continuous clinical outcome measures and changes in these measures were summarised by arm at baseline and at endline as means and standard deviations. Because of the pilot nature of the trial, we did not generate estimates of intervention effect but instead descriptively compare between arms the mean change within the arm of each continuous outcome measure to obtain an indication of the potential for an intervention effect. To help inform a future fully-powered cRCT, we generated preliminary estimates of clustering measured by intracluster correlation coefficients (ICC) of five key outcomes (PHQ-9, WHODAS, GHQ, PCL-5 and RTC). Although in a future trial, we expect that randomisation will occur at the VDC level, it is not possible to obtain ICC estimates for clustering by VDC as only two VDCs are enrolled in this pilot. Instead, we sought to generate estimates of clustering at a smaller unit, namely that of the ward (at baseline) and of the group at endline for participants in the Group PM+ VDC. Such ICC estimates were generated using an intercept-only linear mixed model estimated using restricted maximum likelihood estimation with random intercepts for ward (for baseline data) or for group (for endline data).

The qualitative data were analysed using a thematic content analysis approach. Interviews were first recorded, transcribed verbatim and translated for subsequent analysis. Researchers first familiarised themselves with the transcripts, coded interviews based on previously identified themes and subthemes, added further themes if necessary and finalised coding. Data were then reviewed by code to further draw out key information and quotes were identified that illustrated significant themes.

### Ethics

Ethical approval was obtained from the National Health Research Council (NHRC, reg #371/2016) and the WHO Ethical Review Committee (ERC.0002817). Participants were enrolled only after voluntary written consent (verbal consent only if the participant was illiterate). Participants with suicidal planning were reported immediately to the counsellor for follow-up and all changes in treatment resulting from adverse events or serious adverse events were reported to the Data Safety Management Committee (DSMC). TPO Nepal was responsible for the data collection, storage and making data available to the DSMC, funders and IRBs for audit when necessary.

## Results

### Study population and baseline descriptives

**Fig. 1.**
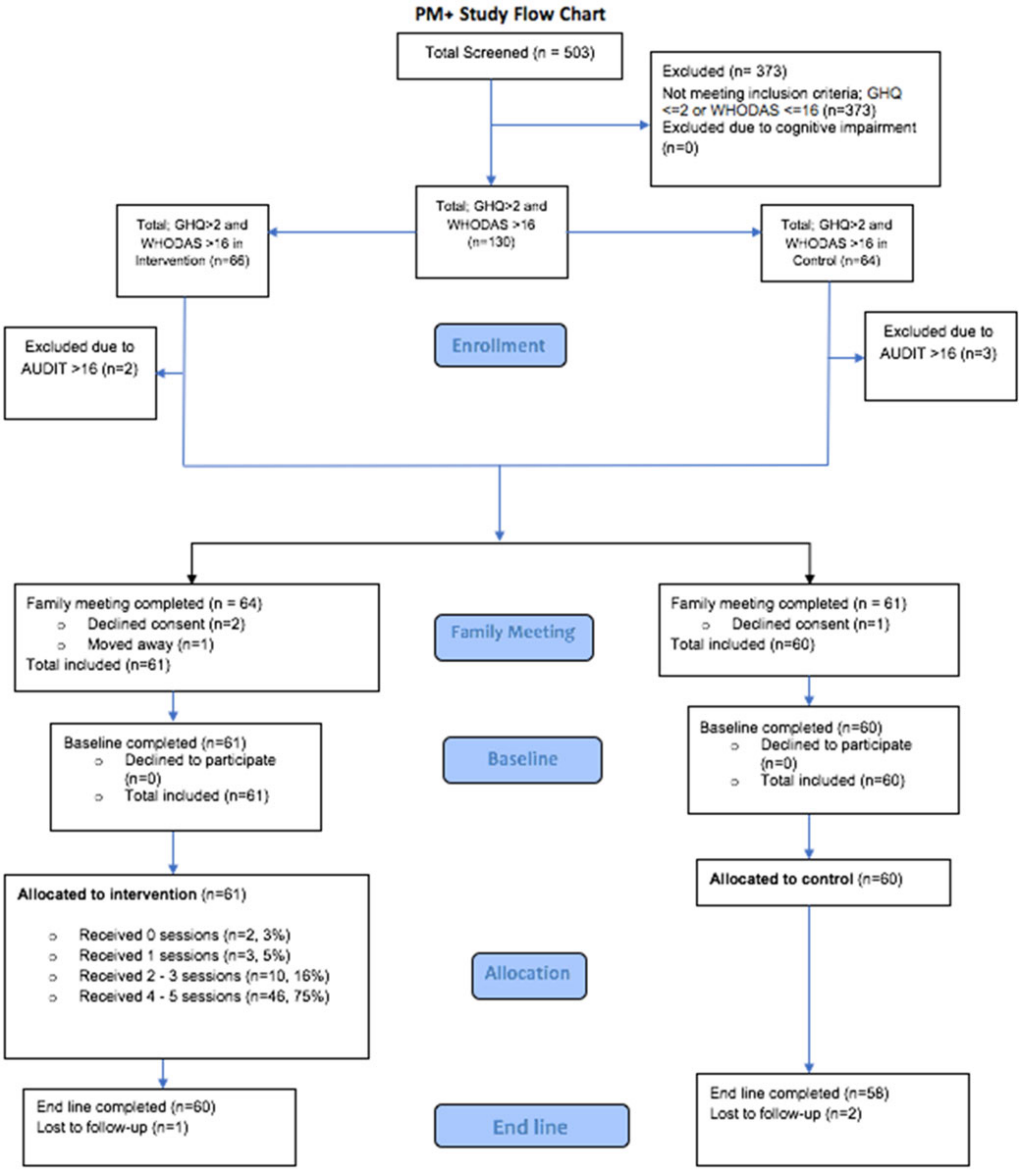
Group PM+ study flow chart.

A total of 130 (25.8%) of the 503 screen individuals were screened positive, of which 66 and 64 were in the Group PM+ VDC and EUC VDC, respectively (Fig. 1). Of these 130 individuals, five were excluded due to an AUDIT score of 16 or more. Of the remaining 125 eligible individuals, all initially consented but there were four further exclusions before baseline. Three participants declined consent to conduct the family meeting and one participant moved away before the family meeting could be conducted. As a result, 121 (24.1%) individuals were eligible and did not withdraw before baseline, of which all (100%) completed the baseline survey. There were ten males in each arm.

### Feasibility and acceptability

This study showed good feasibility with high retention (97.5%) of the 121 participants from baseline to endline. There were no missing items among the five multi-item variables, for the five quantitative outcome measures, for all the 121 participants at baseline. The 118 participants at endline, all of whom had all five key multi-item variables available, had no missing items. Moreover, a majority of (52.5%) participants attended all five group sessions with only five participants (8%) attending fewer than three of the five group sessions (Table 2).

**Table 2.** Quantitative acceptability and feasibility measures

Variable – *n* (%)^a^	Group PM+	EUC
Acceptability of intervention	*n* = 4	*n* = 4
Competency in common factors (%, IQR)		
Pre-training in psychosocial foundations	23% (11–44%)	27% (11–61%)
Post-training in psychosocial foundations	76% (61–89%)	84% (72–94%)
Fidelity of PM+ facilitators		
To 75% or more items in each of 5 group sessions	4 (100%)	**–**
To 75% or more items in more than 3 group sessions	4 (100%)	**–**
Group PM+ participation	*n* = 61	*n* = 60
Number of sessions attended		**–**
0	2 (3%)	**–**
1	3 (5%)	**–**
2	0 (0%)	**–**
3	10 (16.4%)	**–**
4	14 (23.0%)	**–**
5	32 (52.5%)	**–**
		**–**
Outcome measurements	*n* = 61	*n* = 60
All items of outcome measured at baseline^b^		
PHQ-9	61 (100%)	60 (100%)
WHODAS	61 (100%)	60 (100%)
GHQ	61 (100%)	60 (100%)
PCL-5	61 (100%)	60 (100%)
RTC	61 (100%)	60 (100%)
All items of outcome measured at endline^b^^,^^c^		
PHQ-9	58 (100%)	60 (100%)
WHODAS	58 (100%)	60 (100%)
GHQ	58 (100%)	60 (100%)
PCL-5	58 (100%)	60 (100%)
RTC	58 (100%)	60 (100%)
All key outcomes measured^b^		
Baseline	61 (100%)	60 (100%)
Endline^c^	58 (100%)	60 (100%)
Time (days) between: [median (25^th^, 75^th^ percentile)]		
Screening and baseline interview	10 (5, 32)	9 (6, 14)
Baseline interview and endline interview^c^	42 (36, 47)	48 (43, 52)
Adverse events		
Any adverse event^d^	4	2
Serious adverse event	0	1^e^

a Unless otherwise noted.

b Of five key measures: PHQ-9, WHODAS, GHQ, PCL-5 and RTC. Note, at baseline, WHODAS and GHQ were measured at screening and the remaining three measures at the baseline interview. Additionally, there were no missing items for any of the five measures at either time point.

c Of those who were not lost to follow-up (*n* = 58 in intervention and *n* = 60 in control).

d All six were suicidal thoughts.

e Death unrelated to the study.

Ten of the 61 participants of Group PM+ were male, and six of the ten male participants attended all five sessions. Likewise, the fidelity of PM+ facilitators was adequate with all four Group PM+ facilitators adhering to 75% or more items in each of the group sessions they conducted. Regarding competency in common therapeutic factors, ENACT scores for Group PM+ and EUC groups were above 70%; two CPSWs who scored below 70% were dropped after the initial 20-day psychosocial skills training (as described above) (Table 3).

**Table 3.** Demographic characteristics of 121 enrolled participants by study arm

Characteristic – *n* (%)^a^	Group PM+ (*n* = 61)	EUC (*n* = 60)
Male	10 (17%)	10 (16%)
Age (years) – mean (s.d.)	46.7 (14.0)	49.3 (13.6)
Age categories (years)		
<30	3 (5%)	6 (9.8%)
30–<40	13 (21.7%)	11 (18.0%)
40–<50	18 (30.0%)	19 (31.2%)
50–<60	10 (16.7%)	14 (23.0%)
60–<70	11 (18.3%)	7 (11.5%)
70+	5 (8.3%)	4 (6.6%)
Education level		
Illiterate	36 (59%)	48 (80%)
Informal education	11 (18%)	7 (12%)
Primary	6 (10%)	3 (5%)
Secondary	4 (7%)	2 (3%)
Higher secondary	4 (7%)	0 (0%)
University	0 (0%)	0 (0%)
Occupation		
Farmer	20 (33%)	21 (35%)
Office job	2 (3%)	0 (0%)
Business	4 (7%)	0 (0%)
Daily wage labourer	3 (5%)	4 (7%)
Unemployed	1 (2%)	1 (2%)
Housewife	29 (48%)	33 (55%)
Other	2 (4%)	1 (2%)
Marital status		
Unmarried	2 (3%)	2 (3%)
Married	50 (82%)	48 (80%)
Widowed	7 (11%)	9 (15%)
Divorced	1 (2%)	0 (0%)
Separated	1 (2%)	1 (2%)
Family type		
Singular family	26 (43%)	42 (70%)
Nuclear family	35 (57%)	18 (30%)
Lives with		
Extended family with spouse	12 (20%)	16 (27%)
Extended family without spouse	7 (11%)	12 (20%)
With parents	6 (10%)	1 (2%)
Spouse only	6 (10%)	5 (8%)
Spouse and children only	25 (41%)	22 (37%)
Other	5 (8%)	4 (7%)
Number of members in household		
Median (25^th^, 75^th^ percentile)	5 (3, 6)	5 (3, 6)
Caste		
Brahman/Chhetri	13 (21%)	27 (45%)
Dalit	9 (15%)	14 (23%)
Danuwar	23 (38%)	3 (5%)
Other	16 (26%)	16 (26%)
Religion		
Hindu	55 (90%)	52 (87%)
Other	6 (10%)	8 (13%)
Most-used language		
Nepali	37 (61%)	58 (97%)
Danuwar	22 (36%)	2 (3%)
Other	2 (3%)	0 (0%)
Self-perceived socioeconomic status		
Very bad	0 (0%)	11 (18%)
Bad	8 (13%)	17 (28%)
Normal	38 (62%)	27 (45%)
Good	14 (23%)	5 (8%)
Very good	1 (2%)	0 (0%)
Chronic disease		
Reported a chronic disease	18 (30%)	20 (33%)
Of those with chronic disease, primary^b^ type of disease		
Hypertension	5 (28%)	6 (30%)
Asthma	3 (17%)	6 (30%)
Other	10 (55%)	8 (40%)
Earthquake exposure		
Experienced aftershocks	60 (98%)	57 (95%)
Home badly damaged or destroyed	26 (43%)	34 (57%)
Trapped under rubble	8 (13%)	1 (2%)
Injury from the earthquake	4 (7%)	6 (10%)
Close friends or family injured	7 (11%)	5 (8%)
Close friends or family killed	1 (2%)	2 (3%)

a Unless otherwise stated.

b If more than one was reported, the primary type was selected and the secondary reported in ‘Other’. In these data, each person reported at most one.

### Clinical outcomes

At baseline, outcomes were broadly comparable between the participants of the Group PM+ and EUC arms with mean (s.d.) PHQ-9 scores of 9.8 (4.9) and 10.7 (4.4) in the Group PM+ and EUC arms, respectively (Table 4). Across the 121 participants, the PHQ-9 had a mean (s.d.) of 10.3 (4.6). The WHODAS had a mean (s.d.) of 21.3 (4.8), the GHQ-12 had a mean (s.d.) of 22.8 (5.0), the PMHP had a mean (s.d.) of 10.7 (3.0), and the PCL-5 had a mean (s.d.) of 19.5 (6.8). Baseline outcomes for the 118 participants who also had data at endline were comparable to those of the overall study population of 121 participants. For the 118 participants with endline data, nearly all outcomes improved on average over time in both arms, decreases in PHQ-9, GHQ-10, WHODAS, PMPH and PCL in both study arms and an increase in MSPSS in both study arms. For all five key outcomes, the estimated mean improvement was larger in the Group PM+ arm than the EUC arm, with larger mean decreases in scores observed for all five outcomes. No formal between-group comparisons were made given that the pilot trial was not powered to detect meaningful differences. For the other outcomes of RTC and MSPSS, as hypothesised, both increased on average in the Group PM+ arm, whereas very small decreases were observed in the EUC group; of 5.0 (s.d. = 5.8) in Group PM+ compared to an average decrease in EUC of −0.7 (4.6). Estimates of clustering by ward at baseline were large ranging from 0.10 (95% CI 0.03–0.41) for WHODAS to 0.21 (0.08–0.45) for PCL-5 when clustering was by ward at enrolment.

**Table 4.** Outcomes at baseline and endline of *n* = 121 enrolled participants by study arm – mean (s.d.) reported

		For all *n* = 121 enrolled		For *n* = 118 not lost to follow-up at endline						ICC (95% CI)	
		Baseline		Baseline		Endline		Change		Baseline	Endline
Construct	Instrument (range)	Group PM+ (*n* = 61)	EUC (*n* = 60)	Group PM+ (*n* = 60)	EUC (*n* = 58)	Group PM+ (*n* = 60)	EUC (*n* = 58)^a^	Group PM+ (*n* = 60)	EUC (*n* = 58)	(*n* = 121)^b^	(*n* = 60)^c^
Primary outcome											
Depressive symptoms	PHQ-9 (0–27)	9.8 (4.9)	10.7 (4.4)	9.7 (4.8)	10.9 (4.3)	6.2 (3.7)	9.3 (4.3)	−3.5 (4.8)	−1.6 (3.4)	0.12 (0.03, 0.41)	–^d^
Secondary outcomes											
Daily functioning	WHODAS (12–60)	21.8 (5.3)	20.8 (4.1)	21.5 (4.9)	20.9 (4.2)	12.1 (8.0)	15.7 (6.4)	−9.4 (8.4)	−5.2 (6.7)	0.10 (0.01, 0.59)	0.09 (0.01, 0.62)
General psychological distress	GHQ-12 (0–36)	24.3 (4.8)	21.3 (4.7)	24.2 (4.8)	21.4 (4.8)	11.9 (6.6)	17.6 (6.0)	−12.3 (7.5)	−3.7 (7.0)	0.16 (0.02, 0.62)	0.06 (0.00, 0.75)
Psychosocial MH problems	PMHP (5–20)	10.2 (3.3)	11.1 (2.7)	10.1 (3.3)	11.2 (2.7)	9.1 (3.0)	11.2 (2.9)	−1.0 (2.8)	−0.1 (2.7)	0.16 (0.05, 0.41)	0.03 (0.00, 0.97)
PTSD	PCL-5 (8–40)	17.6 (7.2)	21.5 (5.9)	17.5 (7.2)	21.8 (5.7)	14.8 (8.1)	20.5 (5.6)	−2.7 (7.0)	−1.3 (5.6)	0.21 (0.08, 0.45)	–^d^
Other outcomes											
Reducing tension skills	RTC (0–40)	15.6 (4.7)	10.1 (5.0)	15.6 (4.8)	10.2 (5.1)	20.6 (5.8)	9.4 (4.2)	5.0 (5.8)	−0.7 (4.6)	0.24 (0.09, 0.50)	0.21 (0.05, 0.59)
Perceived social support	MSPSS (12–60)	33.4 (7.9)	29.9 (8.7)	33.3 (8.0)	29.6 (8.7)	34.2 (7.0)	29.4 (8.7)	0.9 (7.5)	−0.1 (7.9)	–^d^	0.04 (0.00, 0.87)

MH, mental health.

a Estimated using linear mixed-effects regression with a fixed intercept and random intercepts for the unit of clustering, with estimation by restricted maximum likelihood estimation to account for the small number of clusters.

b Unit of clustering is ward at baseline, of which there are 17.

c Unit of clustering is PM+ group, of which there are 10.

d Not estimable.

Estimates of clustering by group at endline were smaller ranging from 0.03 (95% CI 0–0.97) to 0.09 (0.01–0.62), though were not estimable for PHQ-9 and PCL-5. As expected, confidence intervals were wide in all cases due to the small sample size.

### Qualitative outcomes

As captured by responses from CPSWs and RAs, the study was initially met with hesitancy from community members due to prior notions that only those with severe mental illnesses need support. Referring to mental health issues as ‘man ko samasya’ (heart–mind problems) (Kohrt and Harper, 2008) or ‘tension’ (an English term used commonly in Nepal for distress) (Clarke *et al*., 2014*b*; Rai *et al*., 2018), non-stigmatising idioms of distress made the study more acceptable to community members. CPSWs reported that community sensitisation events helped clarify to the community that this programme was for people with general distress rather than severe mental illness. Group PM+ participants found the Nepali programme name, ‘Khulla Man’ meaning ‘an open and light heart–mind’ as a cultural concept of catharsis, to be acceptable. Both male and female participants also referred to their own heart–mind as being lighter after completion of the programme.

Male and female Group PM+ participants responded positively to the programme. Participants reported enjoying the group format of the programme and spending time outside the home with others. Both male and female participants reported that the group format helped them realise that others experience similar problems and that problems should be shared. They noted improvements in their somatic symptoms, such as restlessness and feelings of weakness, and social functioning. Though session materials such as calendars for reminders seemed to be effective as reported by the facilitators, some participants noted that they were too busy to practise techniques at home but enjoyed the sessions and requested additional weeks. Participants' expectations of monetary incentives, rather than the content of the programme, seemed to have attributed to drop-outs. Facilitators noted that after several rounds of conducting PM+ group sessions, other community members also showed interest in participating in Group PM+.

This was the first Group PM+ study that included males and demonstrated a high retention rate amongst their groups. Male participants also reported enjoying the session activities and case stories, and practised techniques at home. Programme staff reported that barriers to recruiting men included their initial hesitance in discussing personal problems and emotions with others, busy work schedule and lack of men in the villages due to labour migration.

Participants in both arms preferred to conduct assessments with gender-matched RAs due to fear of perceptions from family and community members. Some EUC participants noted that assessments helped them feel lighter and thought of them as the treatment. Others were disappointed by the lack of treatment especially because accessing referral services was a noted challenge. Participants that visited the health post for treatment and were dissuaded when it was closed or did not have the necessary medications. Health workers trained in mhGAP suggested additional refresher trainings to better support referrals (Table 5).

**Table 5.** Qualitative interview results

Domain	Theme	Quote
Acceptability	Idiom usage (usage of ‘khulla man’, use of tension)	‘*We learned that we shouldn't hide our tension and that we need to share it with our friends. We shouldn't let our stress affect us. When we share our feelings with our friends then it will help us a lot. I learned this from ‘*khulla mann*’ program. We learned that we should give suggestions to our neighbors too so I liked it.*’ – Female participant, Group PM+ ‘*I have good thoughts these days, I am satisfied…So this has given me new strength, motivation. I have learned that we should open up about our problems and only then other people will be willing to help us…I received help from my sister-in-laws. If I hadn't opened up (*Khulla*) about my problem and had stayed by myself then who would know about my problem? If we open up about the problem we are facing then they will help in what they can. So I am really happy to be able to learn all these things.*’ – Female participant, Group PM+
	Acceptability of assessments and intervention	‘*I feared that people in the community will say anything bad about it [RA]…because he was a man…my husband isn't here and my mother in-law was also here so I was really stressed about it but I took time to talk to him.*’ – Female participant, Enhanced Usual Care ‘*My child is very small. So I used to be late [to sessions]…when I asked my sister-in-laws to look after my child, they used to take it in a negative way…I had a small store so I had to manage time to go to the program. But it was manageable.*’ �� Female participant, Group PM+ ‘*In this last session that I am conducting, the participants said that they wouldn't be getting anything except lunch so because of this reason, some didn't come.*’ – Facilitator, Group PM+
	Benefits of a group format	‘*It felt like everyone has problems and not just me. I used to think that only I went through things but I asked the others if they also had problems.*’ – Female participant, Group PM+ ‘*I have made friends too. We [participants] live nearby so we meet with each other. We share that the program was good and that we will join such programs again. All of us live nearby so we gather and talk about our problems.*’ – Female participant, Group PM+ Participant
	Hesitancy because of prior notions of MH	‘*Yes people have said negative things about this program too because they haven't understood it. Those who have understood about this program have realized that it is good.*’ – Research assistant, Group PM+ Arm ‘*After learning the skills, it's something you do for yourself. If I share with others, they may say, ‘this program isn't good.’ They might make fun of me…If they say things like that, then it won't feel good for me…It is best for me to learn and just do it myself.*’ – Female participant, Group PM+ ‘*In the beginning, they didn't open up well. When we went for community sensitization in the beginning, no one shared with us that they have mental health problems…And they opened up later about the kind of problems that they were experiencing…they feared to open up at first because…people in the community might say negative things to them.*’ – Community psychosocial worker, Enhanced Usual Care
Perceived utility	Improvements in somatic symptoms	‘*…when I feel weak, I do those activities. Now I have forgotten all [all of tension]. I used to have so much tension. I didn't want to eat. Couldn't sleep. I didn't want to walk anywhere. My legs used to be so sore and tired after I walked…Now I have forgotten all these things.*’ – Female participant, Group PM+
	Session materials/practicing outside of sessions	‘*Whenever I feel bored or bad I look at the calendar [from the program] and I would remember what was taught in the training and I would do it. Before, I didn't want to sit with friends or attend any kind of wedding or pooja (prayer) programs. I just wanted to stay alone and I used to think a lot and weep. But after attending the* Khulla Man *program, I don't feel that way.*’ – Female participant, Group PM+
	Males in Group PM+	‘*Before when we used to have conflict in our family, we used to have lots of stress and we didn't know what to do. But after this training, even though we have conflict in family, we now have realization that we shouldn't hide these things in ourselves but we should rather share it with our close friends…You have to tell it to someone you trust; be it your wife or friends.*’ – Male participant, Group PM+ ‘*I liked everything about this program. The story of Ram Bahadur was shared from the beginning…he felt the same way as us. I learned what might happen to our heart…So we learned how to calm our heart…by reading the story. I realized that I had these kind of problems but there might be other people who might have faced such problems before too.*’ – Male participant, Group PM+

## Discussion

The RCT met all pre-defined feasibility and acceptability criteria (Table 1). The high rates of participation in the sessions indicate that participants found the intervention to be acceptable, which was supported by the qualitative findings. Additionally, only three participants were lost to follow-up which indicates the feasibility of trial procedures. The feasibility of assessments, procedures and the intervention indicates that a fully-powered Group PM+ trial is achievable in the Nepal context.

The descriptive study results, if also supported by the fully-powered trial, suggest better improvements in the Group PM+ arm, especially in daily functioning and general distress, and indicate that Group PM+ delivered by non-specialists has the potential to reduce psychological distress relative to EUC. Though this study is not powered, findings are in line with current evidence that effective psychological interventions can be delivered by non-specialised workers (Singla *et al*., 2017). This was supported by the qualitative analysis in which Group PM+ participants mentioned overall changes in somatic symptoms and an increased understanding of how to manage their problems.

The study was initially met with hesitancy amongst community members due to their understanding that only those with severe mental illnesses need support. This highlights the importance of using de-stigmatising local idioms and language during the initial planning phase with local stakeholders, the recruitment process, assessments and the intervention itself (Kohrt and Harper, 2008; Kohrt and Hruschka, 2010). As experienced by the CPSWs in both arms, sensitisation events worked to normalise experiencing adversity and distress, and to differentiate to the community that this programme was for those with general distress.

Based on the qualitative evaluations, the group format also had some inherent benefits, such as reducing self-stigma since participants felt that many others in their community were also seeking support. Perhaps because most of the Group PM+ participants were housewives, they noted enjoying the company of a group and taking time away from daily household chores. Furthermore, the pervasiveness of community groups (mother's groups, youth groups, etc.) in rural Nepal and other LMICs adds to the acceptability of a group intervention in the Nepal context (Clarke *et al*., 2014*a*).

A strength of this study is the addition of the combined competency and fidelity checklist to measure facilitator competency in common factors and adherence to the manual during intervention delivery. Another strength was the use of the RTC to measure the participant's use of skills learned in Group PM+ sessions. The outcomes evaluation indicates an increase in RTC scores at follow-up in the intervention arm as compared to the control arm, suggesting that the delivery and uptake of intervention strategies appears feasible. However, participants indicated practising some techniques more than others. A recommendation for the definitive trial is to develop and strengthen tools that reinforce the techniques learned in the sessions.

Because the limited number of trained mental health workers is a larger barrier to care in LMICs (Kakuma *et al*., 2011), the referral system was a noted challenge. Though health posts with mhGAP trained health workers were near-by and an improvement from the standard of care in rural Nepal, participants faced barriers such as absence of trained health workers, lack of medication and closed facilities due to the rural nature of the study area. More efforts should be made in the next trial to refer all participants to better-resourced health facilities to ensure follow through, especially for the EUC arm.

This was the first Group PM+ study that included males and demonstrated a high retention rate amongst male groups. Recruiting men was a noted challenge because of labour migration, work outside of the home and their hesitancy in discussing personal problems. The overall feasibility and acceptability of conducting the intervention and EUC procedures amongst men indicates that it is possible to include both genders in a larger trial in the Nepal context, with some potential barriers in recruitment.

Limitations of the study design include the risk of contamination and the inability to maintain complete blinding. The CPSWs and RAs from the two arms were initially trained together. Study sites were close in distance, which may have increased the likelihood of participants, CPSWs or research staff communicating with each other. However, all local staff were assigned to work in their VDC only, which decreased the likelihood of un-blinding. Stricter blinding procedures are recommended for the fully-powered trial.

The initial planning phase with stakeholders, recruitment process, assessments and the Group PM+ intervention itself showed feasibility and acceptability among both male and female participants. The estimated mean improvement was larger, for all key outcome measures, in the intervention compared to the EUC arm. A larger fully-powered trial will seek to establish intervention effectiveness in the Nepal context.

## Supplementary material

For supplementary material accompanying this paper visit http://dx.doi.org/10.1017/S2045796020000414. click here to view supplementary material

## Data

Raw data are available as additional supporting files.

## References

[ref1] Ashworth M, Shepherd M, Christey J, Matthews V, Wright K, Parmentier H, Robinson S and Godfrey E (2004) A client-generated psychometric instrument: the development of ‘PSYCHLOPS’. Counselling and Psychotherapy Research 4, 27–31.

[ref2] Bryant RA, Schafer A, Dawson KS, Anjuri D, Mulili C, Ndogoni L, Koyiet P, Sijbrandij M, Ulate J, Harper Shehadeh M, Hadzi-Pavlovic D and van Ommeren M (2017) Effectiveness of a brief behavioural intervention on psychological distress among women with a history of gender-based violence in urban Kenya: a randomised clinical trial. PLoS Medicine 14, 1–20. 10.1371/journal.pmed.1002371 PMC5557357 28809935

[ref3] Clarke K, Azad K, Kuddus A, Shaha S, Nahar T, Aumon BH, Hossen MM, Beard J, Costello A, Houweling TA, Prost A and Fottrell E (2014 *a*) Impact of a participatory intervention with women's groups on psychological distress among mothers in rural Bangladesh: secondary analysis of a cluster-randomised controlled trial. PLoS ONE 9, 1–8. 10.1371/journal.pone.0110697 PMC4199763 25329470

[ref4] Clarke K, Saville N, Bhandari B, Giri K, Ghising M, Jha M, Jha S, Magar J, Roy R, Shrestha B, Thakur B, Tiwari R, Costello A, Manandhar D, King M, Osrin D and Prost A (2014 *b*) Understanding psychological distress among mothers in rural Nepal: a qualitative grounded theory exploration. BMC Psychiatry 14, 60. 24581309 10.1186/1471-244X-14-60 PMC3943437

[ref5] Dawson KS, Bryant RA, Harper M, Kuowei Tay A, Rahman A, Schafer A and van Ommeren M (2015) Problem Management Plus (PM+): a WHO transdiagnostic psychological intervention for common mental health problems. World Psychiatry 14, 354–357. 26407793 10.1002/wps.20255 PMC4592660

[ref6] Dawson KS, Schafer A, Anjuri D, Ndogoni L, Musyoki C, Sijbrandij M, van Ommeren M and Bryant RA (2016) Feasibility trial of a scalable psychological intervention for women affected by urban adversity and gender-based violence in Nairobi. BMC Psychiatry 16, 410. 27863515 10.1186/s12888-016-1117-x PMC5116169

[ref7] Hendrickson ZM, Kim J, Tol WA, Shrestha A, Kafle HM, Luitel NP, Thapa L and Surkan PJ (2018) Resilience among Nepali widows after the death of a spouse: ‘that was my past and now I have to see my present’. Qualitative Health Research 28, 466–478. 29110564 10.1177/1049732317739265

[ref8] Jordans MJ and Tol WA (2013) Mental health in humanitarian settings: shifting focus to care systems. International Health 5, 9–10. 24029839 10.1093/inthealth/ihs005

[ref10] Jordans MJ, Kohrt BA, Luitel NP, Komproe IH and Lund C (2015) Accuracy of proactive case finding for mental disorders by community informants in Nepal. British Journal of Psychiatry 207, 501–506. 26450582 10.1192/bjp.bp.113.141077 PMC4664856

[ref11] Jordans MJ, Luitel NP, Pokhrel P and Patel V (2016) Development and pilot testing of a mental healthcare plan in Nepal. British Journal of Psychiatry 208, 21–28. 10.1192/bjp.bp.114.153718 PMC4698553 26447173

[ref12] Kakuma R, Minas H, van Ginneken N, Dal Poz MR, Desiraju K, Morris JE, Saxena S and Scheffler RM (2011) Human resources for mental health care: current situation and strategies for action. Lancet (London, England) 378, 1654–1663. 10.1016/S0140-6736(11)61093-3 22008420

[ref13] Kane JC, Luitel NP, Jordans MJD, Kohrt BA, Weissbecker I and Tol WA (2018) Mental health and psychosocial problems in the aftermath of the Nepal earthquakes: findings from a representative cluster sample survey. Epidemiology and Psychiatric Sciences 27, 301–310. 28065208 10.1017/S2045796016001104 PMC5502203

[ref14] Khan MN, Hamdani SU, Chiumento A, Dawson K, Bryant RA, Sijbrandij M, Nazir H, Akhtar P, Masood A, Wang D, Wang E, Uddin I, van Ommeren M and Rahman A (2019) Evaluating feasibility and acceptability of a group WHO trans-diagnostic intervention for women with common mental disorders in rural Pakistan: a cluster randomised controlled feasibility trial. Epidemiology and Psychiatric Sciences 28, 77–87. 28689511 10.1017/S2045796017000336 PMC6998939

[ref15] Kohrt BA and Harper I (2008) Navigating diagnoses: understanding mind-body relations, mental health, and stigma in Nepal. Culture, Medicine and Psychiatry 32, 462–491. 10.1007/s11013-008-9110-6 PMC3869091 18784989

[ref16] Kohrt BA and Hruschka DJ (2010) Nepali concepts of psychological trauma: the role of idioms of distress, ethnopsychology and ethnophysiology in alleviating suffering and preventing stigma. Culture, Medicine and Psychiatry 34, 322–352. 10.1007/s11013-010-9170-2 PMC3819627 20309724

[ref17] Kohrt BA, Hruschka DJ, Worthman CM, Kunz RD, Baldwin JL, Upadhaya N, Acharya NR, Koirala S, Thapa SB, Tol WA, Jordans MJ, Robkin N, Sharma VD and Nepal MK (2012) Political violence and mental health in Nepal: prospective study. British Journal of Psychiatry 201, 268–275. 22878131 10.1192/bjp.bp.111.096222 PMC3461445

[ref18] Kohrt BA, Jordans MJ, Koirala S and Worthman CM (2015 *a*) Designing mental health interventions informed by child development and human biology theory: a social ecology intervention for child soldiers in Nepal. American Journal of Human Biology 27, 27–40. 25380194 10.1002/ajhb.22651 PMC5483323

[ref19] Kohrt BA, Jordans MJ, Rai S, Shrestha P, Luitel NP, Ramaiya MK, Singla DR and Patel V (2015 *b*) Therapist competence in global mental health: development of the ENhancing Assessment of Common Therapeutic factors (ENACT) rating scale. Behaviour Research and Therapy 69, 11–21. 25847276 10.1016/j.brat.2015.03.009 PMC4686771

[ref20] Kohrt BA, Worthman CM, Ressler KJ, Mercer KB, Upadhaya N, Koirala S, Nepal MK, Sharma VD and Binder EB (2015 *c*) Cross-cultural gene-environment interactions in depression, post-traumatic stress disorder, and the cortisol awakening response: FKBP5 polymorphisms and childhood trauma in South Asia. International Review of Psychiatry 27, 180–196. 26100613 10.3109/09540261.2015.1020052 PMC4623577

[ref21] Kohrt BA, Luitel NP, Acharya P and Jordans MJ (2016) Detection of depression in low resource settings: validation of the Patient Health Questionnaire (PHQ-9) and cultural concepts of distress in Nepal. BMC Psychiatry 16, 58. 26951403 10.1186/s12888-016-0768-y PMC4782581

[ref24] Luitel NP, Jordans MJ, Sapkota RP, Tol WA, Kohrt BA, Thapa SB, Komproe IH and Sharma B (2013) Conflict and mental health: a cross-sectional epidemiological study in Nepal. Social Psychiatry and Psychiatric Epidemiology 48, 183–193. 22777395 10.1007/s00127-012-0539-0

[ref25] Luitel NP, Jordans MJ, Adhikari A, Upadhaya N, Hanlon C, Lund C and Komproe IH (2015) Mental health care in Nepal: current situation and challenges for development of a district mental health care plan. Conflict and Health 9, 3. 25694792 10.1186/s13031-014-0030-5 PMC4331482

[ref26] Minhas FA and Mubbashar MH (1996) Validation of General Health Questionnaire (GHQ-12) in primary care settings of Pakistan. Journal of College of Physicians and Surgeons Pakistan 6, 133–136.

[ref27] Neacsiu AD, Rizvi SL, Vitaliano PP, Lynch TR and Linehan MM (2010) The dialectical behavior therapy ways of coping checklist: development and psychometric properties. Journal of Clinical Psychology 66, 563–582. 20455249 10.1002/jclp.20685

[ref28] Patel V, Saxena S, Lund C, Thornicroft G, Baingana F, Bolton P, Chisholm D, Collins PY, Cooper JL, Eaton J, Herrman H, Herzallah MM, Huang Y, Jordans MJD, Kleinman A, Medina-Mora ME, Morgan E, Niaz U, Omigbodun O, Prince M, Rahman A, Saraceno B, Sarkar BK, De Silva M, Singh I, Stein DJ, Sunkel C and UnUtzer J (2018) The Lancet Commission on global mental health and sustainable development. Lancet (London, England) 392, 1553–1598. 10.1016/S0140-6736(18)31612-X 30314863

[ref29] Price M, Szafranski DD, van Stolk-Cooke K and Gros DF (2016) Investigation of abbreviated 4 and 8 item versions of the PTSD Checklist 5. Psychiatry Research 239, 124–130. 27137973 10.1016/j.psychres.2016.03.014

[ref30] Rahman A, Khan MN, Hamdani SU, Chiumento A, Akhtar P, Nazir H, Nisar A, Masood A, Din IU, Khan NA, Bryant RA, Dawson KS, Sijbrandij M, Wang D and van Ommeren M (2019) Effectiveness of a brief group psychological intervention for women in a post-conflict setting in Pakistan: a single-blind, cluster, randomised controlled trial. Lancet (London, England) 393, 1733–1744. 10.1016/S0140-6736(18)32343-2 30948286

[ref31] Rai S, Gurung D, Kaiser BN, Sikkema KJ, Dhakal M, Bhardwaj A, Tergesen C and Kohrt BA (2018) A service user co-facilitated intervention to reduce mental illness stigma among primary healthcare workers: utilizing perspectives of family members and caregivers. *Families*, Systems and Health 36, 198–209. 10.1037/fsh0000338 PMC6005191 29902036

[ref32] Sangraula M, Van't Hof E, Luitel NP, Turner EL, Marahatta K, Nakao JH, van Ommeren M, Jordans MJD and Kohrt BA (2018) Protocol for a feasibility study of group-based focused psychosocial support to improve the psychosocial well-being and functioning of adults affected by humanitarian crises in Nepal: Group Problem Management Plus (PM+). Pilot and Feasibility Studies 4, 126. 30038793 10.1186/s40814-018-0315-3 PMC6052626

[ref33] Schwartz AC, Bradley RL, Sexton M, Sherry A and Ressler KJ (2005) Posttraumatic stress disorder among African Americans in an inner city mental health clinic. Psychiatric Services 56, 212–215. 15703352 10.1176/appi.ps.56.2.212

[ref34] Singla DR, Kohrt BA, Murray LK, Anand A, Chorpita BF and Patel V (2017) Psychological treatments for the world: lessons from low- and middle-income countries. Annual Review of Clinical Psychology 13, 149–181. 10.1146/annurev-clinpsy-032816-045217 PMC5506549 28482687

[ref35] Subba P, Luitel NP, Kohrt BA and Jordans MJD (2017) Improving detection of mental health problems in community settings in Nepal: development and pilot testing of the community informant detection tool. Conflict and Health 11, 28. 29181088 10.1186/s13031-017-0132-y PMC5694900

[ref36] Thapa SB and Hauff E (2012) Perceived needs, self-reported health and disability among displaced persons during an armed conflict in Nepal. Social Psychiatry and Psychiatric Epidemiology 47, 589–595. 21476014 10.1007/s00127-011-0359-7 PMC3304067

[ref37] Thornicroft G, Chatterji S, Evans-Lacko S, Gruber M, Sampson N, Aguilar-Gaxiola S, Al-Hamzawi A, Alonso J, Andrade L, Borges G, Bruffaerts R, Bunting B, de Almeida JM, Florescu S, de Girolamo G, Gureje O, Haro JM, He Y, Hinkov H, Karam E, Kawakami N, Lee S, Navarro-Mateu F, Piazza M, Posada-Villa J, de Galvis YT and Kessler RC (2017) Undertreatment of people with major depressive disorder in 21 countries. British Journal of Psychiatry 210, 119–124. 27908899 10.1192/bjp.bp.116.188078 PMC5288082

[ref38] Tol WA, Komproe IH, Jordans MJ, Thapa SB, Sharma B and De Jong JT (2009) Brief multi-disciplinary treatment for torture survivors in Nepal: a naturalistic comparative study. International Journal of Social Psychiatry 55, 39–56. 19129325 10.1177/0020764008091525

[ref39] Tol WA, Kohrt BA, Jordans MJ, Thapa SB, Pettigrew J, Upadhaya N and de Jong JT (2010) Political violence and mental health: a multi-disciplinary review of the literature on Nepal. Social Science and Medicine 70, 35–44. 19833427 10.1016/j.socscimed.2009.09.037

[ref40] Tonsing K, Zimet GD and Tse S (2012) Assessing social support among South Asians: the multidimensional scale of perceived social support. Asian Journal of Psychiatry 5, 164–168. 22813661 10.1016/j.ajp.2012.02.012

[ref41] Zimet GD, Powell SS, Farley GK, Werkman S and Berkoff KA (1990) Psychometric characteristics of the multidimensional scale of perceived social support. Journal of Personality Assessment 55, 610–617. 2280326 10.1080/00223891.1990.9674095

