# Supplementary material for: Feasibility of Group Problem Management Plus (PM+) to improve mental health and functioning of adults in earthquake-affected communities in Nepal
Source: Epidemiol Psychiatr Sci. 2020 May 26;29:e130. doi: 10.1017/S2045796020000414 (PMC7264859; doi:10.1017/S2045796020000414)
Supplement: Supplementary file 1 [file S2045796020000414sup001.docx]

**Supplementary Material**

***Setting***

The two VDCs have a diverse population with over 15 ethnicities. There are no specialized mental health treatment facilities in Sindhuli district, and the closest psychiatric referral services are approximately 6-hours away from the study sites. A functioning, though often not well-resourced, government health post in each VDC is the first portal of care and was used in the study as a point of referral.

***Randomization***

A randomization procedure was used, in which names of the two VDCs were written on cards and placed into a hat. The District Public Health Officer (DPHO) was to draw one card from the hat which would be allocated as the intervention arm VDC and the VDC in the remaining card would be allocated to the control arm. Program staff, including community psychosocial workers (CPSWs) and research assistants (RAs) were only assigned to either VDC after the random drawing to reduce risk of unblinding.

***Recruitment and Training of Non-specialist Providers and Research Assistants***

Five non-specialists, CPSWs as described above, in each arm were hired to facilitate recruitment through community sensitization, conduct family meetings (both arms), and facilitate Group PM+ sessions (treatment arm). Local community members were recruited as non-specialists, as a strategy to increase mental health capacity building in rural areas. Selected CPSWs had at least ten years of education, over 25 years of age, and were living in either of the study VDCs.

CPSWs, in both the control and intervention arms, were first trained for 20 days on basic psychological skills to become CPSWs, which is the standard length of training provided by TPO Nepal throughout the country (Jordans *et al.*, 2003, Kohrt *et al.*, 2015a). This was also based on the expectation, as gathered through the contextualization process, that a briefer training would not equip facilitators to provide quality care to intervention participants. Competency in common factors, a set of therapeutic skills and competencies that are common to different psychological treatments, was assessed with the ENACT (Kohrt *et al.*, 2015b) before and after the core foundational training prior to Group PM+. After the foundational training, competency in at least 70% of the items was needed to be included in the subsequent care provision in Group PM+ and EUC arms. This minimum competency was determined based on prior studies using the ENACT in Nepal (Kohrt *et al.*, 2018).

Subsequently, CPSWs from the intervention arm received an additional 10-day training on Group PM+ using the adapted manual and other clinical materials (such as posters and case stories used in the sessions) to become Group PM+ facilitators. Group “helpers” received a basic 2-day training on assisting facilitators during Group PM+ sessions and participating alongside facilitators in practice PM+ groups. The helper’s role was to encourage participants to attend sessions, support facilitator with group logistics such as arranging materials, setting up the venue and distributing snacks.

Eight Research Assistants (RAs) were also locally recruited from the two sites.

***Blinding***

CPSWs, RAs, trial participants, and local mhGAP trained health workers were blinded to the allocation of the study conditions. The VDCs of the two arms were separated by another VDC which worked as a buffer and physical barrier against contamination between the two arms. CPSWs in both arms were instructed not to disclose the treatment that any participants received except with their clinical supervisors. The trial statistician was blinded to treatment arm during analysis.

***Measures***

Assessments were conducted with participants during baseline (two weeks after screening and before the intervention), and the follow-up assessments were conducted at approximately 8-8.5 weeks after the baseline assessment so that, in the Group PM+ arm, it would be 1 – 1.5 weeks after the last session.

***Screening***

Residents of the study VDCs 18 years of age and older were eligible for enrollment. There was no maximum age for enrollment but assessors used their discretion to discontinue screening for those that were unable to properly comprehend the questions due to age or hearing and speaking ability. Inclusion criteria for the study participants were: score of >2 on the General Health Questionnaire (GHQ) (Minhas & Mubbashar, 1996), and a score of >16 on the WHO Disability Assessment Schedule 2.0 (WHODAS), a questionnaire for functional impairment. The WHODAS has been previously used in Nepal (Thapa & Hauff, 2012, Tol *et al.*, 2010, Tol *et al.*, 2009)) , with high internal consistency between items (α = 0.90) and validity with multiple mental health measures for PTSD (r= 0.37, p < 0.001), depression (r = 0.70, p<0.001), and anxiety (r=0.64, p<0.001). The GHQ-12 has been clinically validated in Nepal (Koirala *et al.*, 1999).

Participants presenting symptoms of psychosis and severe cognitive impairment were excluded from the study. Assessment of this exclusion criteria was based on judgment of the assessors (RAs), who were given training on the exclusion criteria using the CIDT for psychosis (Jordans *et al.*, 2015). If during the screening process, the respondent was not able to comprehend or answer the consent and/or demographic questions coherently, the questionnaire was terminated at that point and the participant was excluded.

The WHO’s guidelines report for the Alcohol Use Disorders Identification Test (AUDIT) that people who score 16 or higher on it are recommended to receive advice plus counseling and continued monitoring (Babor *et al.*, 2001). Therefore, persons with a score >16 on the AUDIT, which assesses alcohol dependency, were excluded from participation and were referred to the mhGAP trained health professional in their VDC, for further continuous support and monitoring.

Imminent risk of suicide was determined through a short screening questionnaire and persons with current suicidal plans and/or current suicidal ideation and prior attempts were referred to a psychosocial counselor but were not excluded from participating in the study.

***Details on Outcomes of Quantitative Indicators***

All Group PM+ facilitators adhered to over 75% of the intervention elements. The two study areas were similar in how affected they were by the earthquake and almost all demographic measures including occupation of participants, religious affiliation, and marital status. Still, there were some differences in the caste composition, primary language of recruited participants, and self-perceived socioeconomic status. This pilot trial was conducted in a small catchment area and differences in socio-demographics will likely not pose as a barrier in a fully powered trial. Though 32 participants (52.5%) attended all 5 sessions, 75% of the participants attended 4 – 5 sessions. This relatively high retention rate can be attributed to; (i) helpers reminding participants, and (ii) facilitators having been recruited from the local area that were able to relate well to their participants.

There were no missing items across the five key outcomes. Fewer than 10% (6 adverse events, and 1 serious adverse event) of adverse events were reported amongst the participants in either the control or intervention arm and there was one death - unrelated to the study, indicating that study procedures and PM+ did not cause harm or exacerbate distress.

**Mechanisms of Action of Group PM+ intervention**

| **PM+ Mechanisms of Action** | **Description of mechanism** | **Potential Impact on Mental Health** | **Implementation of mechanism** |
| --- | --- | --- | --- |
| *Stress Management* | Participants learn deep breathing. They are encouraged to incorporate this mechanism into daily life (i.e. when doing housework, walking, etc.). Grounding techniques are incorporated to bring participants back to the present. | Stress reduction techniques is argued to decrease stress responses | Session 1 |
| *Behavioral Activation* | Participants review the inactivity cycle. They choose a small activity that they enjoy doing (i.e. making and drinking tea, meeting a friend etc.) and create a detailed plan about when and how to conduct this activity as a first step in breaking the inactivity cycle. | Increasing activity has shown to reduce depression | Session 2 |
| *Managing Problems* | Participants learn which of their problems are solvable and which are unsolvable. One problem is chosen amongst the solvable problems and participants brainstorm tangible solutions, then creating manageable steps to accomplish their goals. | Developing the capacity to problem solve is shown to be effective in improving mental health, especially in post-adversity settings | Session 3 |
| *Strengthening Social Support* | Participants learn to recognize who amongst their family and friends are existing and potential sources of support and how best to strengthen connections with them. Social network mapping activities are incorporated in this mechanism. | Receiving social support has demonstrated decreases in stress responses | Session 4 |
| *Consolidation of skills and planning for the future* | Participants review the skills from prior sessions and learn to consolidate the different skills , as well as plan for applying them after conclusion of Group PM+ | Implementation of skills in daily life | Session 5 |

Note: PM+ = Group Problem Management Plus

**Quantitative outcome measures**

| **Construct** | **Instrument** | **Description** |  | | **Assessment Time Periods** | | |
| --- | --- | --- | --- | --- | --- | --- | --- |
|  |  |  |  |  | **Enrollment(**-***t_1_***) | **Baseline** (t_0_) | **Endline** (t_1_)* |
| ***Primary Outcome (Participants)*** | | | | | | | |
| Depression symptoms | *Patient Health Questionnaire (PHQ-9)* | Participants rate depression symptoms over past two weeks |  |  | | X | X |
| ***Secondary Outcomes (Participants)*** | | | | | | | |
| Daily Functioning | *WHODAS* | Participants rate their ability to engage in daily activities |  | X | |  | X |
| General Psychological Distress | *General Health Questionnaire(GHQ-12)* | Participants measure their general psychological distress |  | X | |  | X |
| General Psychological Distress | *Heart-mind* | Participants note if they have had any *“*man ko samasya*”* or heart-mind problems recently |  | X | | X | X |
| Suicidality | *Suicidality* | Participants rate if they have recently had suicidal thoughts, ideation, and plans |  | X | |  |  |
| Alcohol use disorder | *Alcohol Use Disorders Identification Test (AUDIT)* | Participants rate alcohol use and associated behavior, as well as daily ethanol consumption |  | X | |  |  |
| Post-traumatic stress symptoms | *Post-traumatic Stress Disorder Checklist DSM-5 (PCL-5)* | Participants rate their post-traumatic stress symptoms on a scale |  |  | | X | X |
| General Psychological Distress | *Psychosocial Mental Health Problems (PMHP)* | Participants rate their somatic symptoms related to psychosocial health |  |  | | X | X |
| ***Other Outcomes (Participants)*** | | | | | | | |
| Perceived Social Support | *Multidimensional Scale of Perceived Social Support (MSPSS)* | Participants assess their own connectedness with close family, friends and other forms of support |  |  | | X | X |
| Reducing tension skills | *Reducing Tension Checklist (RTC)* | Participants assess their own behavioral and psychosocial skills related to coping |  |  | | X | X |
| Traumatic Events | *Traumatic Events Inventory (TEI)* | Participants rate if they have been exposed to certain traumatic events throughout their lifetime |  |  | | X | X |
| Personalized Outcome | *Psychological Outcomes Profile (PSYCHLOPS)* | Participants list their emotional and practical problems and rate how much each problem affects them |  |  | | X | X |
| * At 8-8.5 weeks after baseline, i.e. 1-1.5 weeks after the final Group PM+ session for intervention participants | | | | | | |  |

**References:**

**Babor TF, Higgins-Biddle JC, Saunders JB and Monteiro MG** (2001) The alcohol use disorders identification test: guideline for use in primary care. World Health Organization, 2nd edition.

**Jordans MJ, Tol WA, Sharma B and van Ommeren M** (2003) Training psychosocial counselling in Nepal: content review of a specialised training programme. *Intervention* **1**, 18–35.

**Kohrt BA, Mutamba BB, Luitel NP, Gwaikolo W, Onyango Mangen P, Nakku J, Rose K, Cooper J, Jordans MJD and Baingana F** (2018) How competent are non-specialists trained to integrate mental health services in primary care? Global health perspectives from Uganda, Liberia, and

Nepal. *International Review of Psychiatry* **30**, 182–198.

**Koirala N, Regmi S, Sharma V and Khalid A** (1999) Sensitivity and validity of the General Health Questionnaire (GHQ-12) in a rural community setting in Nepal. *Journal of Psychiatrists’ Association of Nepal* **1**, 34–40.
